# Supplementary material for: Heterologous Expression of a Glycine soja C2H2 Zinc Finger Gene Improves Aluminum Tolerance in Arabidopsis
Source: Int J Mol Sci. 2020 Apr 15;21(8):2754. doi: 10.3390/ijms21082754 (PMC7215988; doi:10.3390/ijms21082754)
Supplement: Supplementary file 1 [file ijms-21-02754-s001.pdf]

Supplementary Materials:

Table S1. Primers used in the experiment.

Primer Name	Primer Sequence (5'→3')
GsGIS3-F	TCTAGATGGAAACCACCCAACTTGACTACA
GsGIS3-R	GAATTCTCAAGCCGAGCCGAGAGATG
Actin3-F	GCACCACCGGAGAGAAAATA
Actin3-R	GTGCACAATTGATGGACCAG
Tubulin-F	ATCGATTCCGTTCTCGATGT
Tubulin-R	ATCCAGTTCCTCCTCCCAAC
qGIS3-F	CTCTTCGGTTTCAGCGTACA
qGIS3-R	GGGAAGTGGAAGAAGTGACC
ALMT1-F	GGCCGACCGTGCTATACGAG
ALMT1-R	CATGAGTCCTGTGAACTCCC
ALS3-F	TATCGATCCTTGCCGGGACTTCA
ALS3-R	GCTTGTCTTGGCGTTGCTCCTA
GA20OX1-F	TGTGGAAAATCAATGGCGCTC
GA20OX1-R	TCGTTCGATAGAGCCATGAAA
GA3OX1-F	TCGCTGACCCCAAGTGAAT
GA3OX1-R	GCAAATTGTGTCTAAATCTCATCTG
GID1-F	CGACTTGCAAATTCTCGTCCT
GID1-R	AGCAACAAACTTACTCTGCTCTC

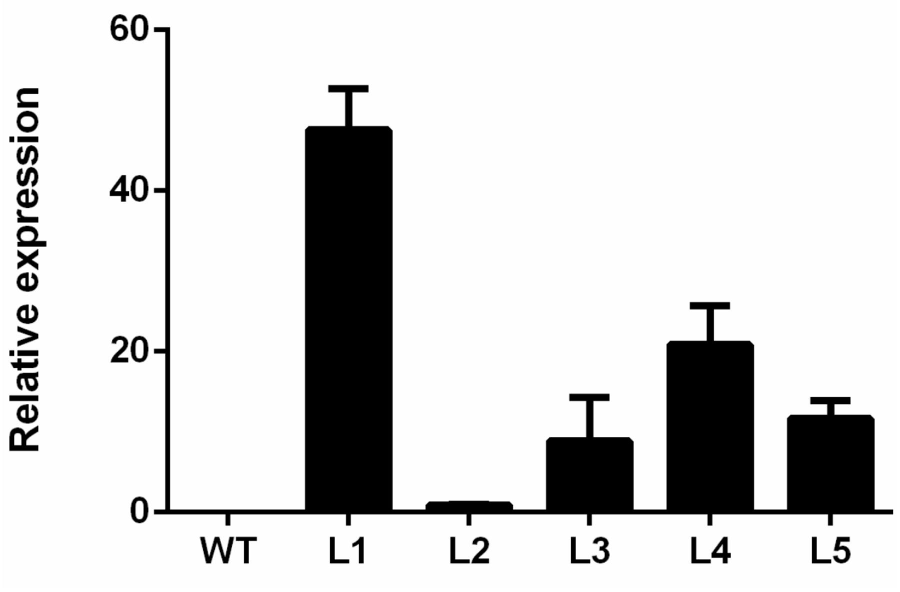

Figure S1: Molecular Identification of Transgenic lines
